# Understanding Biotic Constraints to Taro (*Colocasia esculenta*) Production in the Derived Savanna and Humid Forest Agroecosystems of Nigeria

**DOI:** 10.3390/plants14223457

**Published:** 2025-11-12

**Authors:** Joy Jesumeda Oladimeji, Ranjana Bhattacharjee, Ayodeji Abe, Bolaji Osundahunsi, Ramesh Raju Vetukuri, P. Lava Kumar

**Affiliations:** 1 Plant Breeding Program, Pan African University Life and Earth Sciences Institute (Including Health and Agriculture), Ibadan 200284, Nigeria; 2 International Institute of Tropical Agriculture, PMB 5320, Oyo Road, Ibadan 200001, Nigeria; r.bhattacharjee@cgiar.org (R.B.); bolajiosundahunsi@gmail.com (B.O.); 3 Department of Crop and Horticultural Sciences, University of Ibadan, Ibadan 200284, Nigeria; ayodabe@yahoo.com; 4 Department of Plant Breeding, Swedish University of Agricultural Sciences, Alnarp, SE-23422 Lomma, Sweden; Ramesh.vetukuri@slu.se

**Keywords:** *Colocasia esculenta*, corm-borne diseases, dasheen mosaic virus, taro leaf blight

## Abstract

Taro (*Colocasia esculenta*) is a socioeconomically and nutritionally important crop that is predominantly cultivated in the derived savanna and humid forest agroecosystems of Nigeria. Taro production in the country has declined since the taro leaf blight (TLB) outbreak caused by *Phytophthora colocasiae* Raciborski. This study conducted field surveys during the 2021–2022 production season to assess the status of taro diseases, as well as a structured questionnaire to capture farmers’ management practices and the socio-economic determinants of taro cultivation across seven major taro-producing states in Nigeria. Data was collected from 63 randomly selected farmers across 53 farms, and 449 corms were sampled from farms and markets to assess corm-borne diseases. Sixty-three percent of farmers identified biotic constraints as the major production challenge, with TLB recognized as the most significant threat. Virus-symptomatic plants were not observed in the farmers’ fields, but the occurrence of Dasheen mosaic virus (or *Potyvirus dasheenis*) (DsMV, genus *Potyvirus*) was detected among the plants regenerated from corms collected from farms and markets. The widespread occurrence of TLB and DsMV suggests that these two pathogens pose a serious threat to taro production and that there is a risk of further spread through the continuous recycling of self-sourced planting materials across seasons.

## 1. Introduction

Taro (*Colocasia esculenta* (L.) Schott) is a perennial monocot crop that is often grown as an annual in tropical and subtropical countries [1,2,3]. Although its origin is in the Indo-Malayan region of Southeast Asia [4], taro has been widely cultivated in sub-Saharan Africa (SSA) for several centuries. It has become an important food security crop in the region and is mainly grown as a mixed crop in farms and backyards [3,5,6]. Compared to other root and tuber crops, such as cassava, yams, and sweetpotato, taro corms and leaves are rich in protein with highly digestible starch, rich in minerals such as calcium, potassium, iron, and magnesium, and are a source of vitamin E, C, and B complex [7,8,9,10,11,12,13,14]. Taro is propagated vegetatively using a variety of planting materials, including side shoots, corms, and cormels sourced from their farms or neighboring farms [13,15].

**Figure 1:**
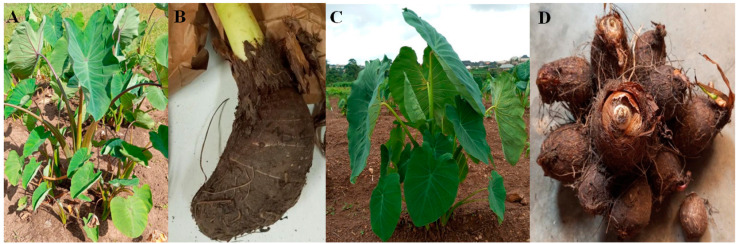
Dasheen taro plant (**A**) and corm (**B**), and the Eddoe taro plant (**C**) and the corm/cormels (**D**).

Nigeria is the world’s largest producer of taro, cultivating it on 1.3 million hectares and producing 8.3 million tons, accounting for 56.2% of the global area harvested and 46.1% of total global production [16]. However, taro yield remains far below the global average of 7.5 tons/ha [16], which can be attributed to a lack of improved varieties, disease susceptibility, and poor corm quality, among others [17]. Both Dasheen (*Colocasia esculenta* var. *esculenta*) (Figure 1A,B) and Eddoe (*C. esculenta* var. *antiquorum*) (Figure 1C,D) types are cultivated in Nigeria [5,18] across six agroecosystems: derived savanna, humid forest, southern guinea savanna, northern guinea savanna, mid-altitude savanna, and arid/semi-arid [1]. However, most taro production is concentrated in the derived savanna and humid forest agroecosystems [1,14]. In the Southeast and South-South regions of Nigeria, the Eddoe type is more prominent and holds significant cultural value, playing an essential role in the local diet and festivals [2]. In the Southwest, the Eddoe type is cultivated for income, and the Dasheen type is mainly grown for consumption.

In 2009, a taro leaf blight (TLB) disease epidemic caused by *Phytophthora colocasiae* Raciborski struck taro farms in Nigeria [1,19], which was also the first official report in Africa. *Phytophthora colocasiae* is an oomycete from the genus *Phytophthora* [20] www.phytophthoradb.org/ (19 February 2021). *P. colocasiae* was first identified in Java by Raciborski in 1900 and is known to be distributed throughout tropical regions worldwide [21,22,23,24]. The pathogen thrives in environments where the day and night temperatures are within 25–28 °C and 20–22 °C, respectively [1,22]. *P. colocasiae* reproduces asexually through sporangiophores, which are short-lived in the infected tissues but perennate in the soil in encysted form, serving as a source of inoculum [25,26,27,28]. The symptomatic plant develops fluid-filled lesions of ~1.5 cm in diameter around the leaf edges. As the disease progresses, the lesions enlarge and develop brown or purple zonate spots. The infected leaf tissues collapse after 20 days, unlike the healthy ones, which can take up to 40 days [27]. Several control methods include the use of fungicides, planting distance, sanitation, crop rotation, and host plant resistance [22,29,30,31,32].

The 2009 outbreak of TLB in Nigeria had a significant negative impact on crop production. In 2009, taro yield and production were 6287 kg/ha and 3.03 million tons [16]. However, from 2010, yields began to decline, falling to 5685 kg/ha in 2010 and 4910 kg/ha by 2012 [16]. This decline occurred despite an expansion in harvested area, demonstrating that farmers attempted to compensate for the losses by cultivating more land, but productivity per hectare continued to suffer. By 2016, the production area had increased and returned to pre-TLB epidemic levels [16]. Taro is an orphan crop, and no improved variety is available in the country, resulting in farmers shifting to other crops. A similar trend was reported following the TLB outbreak in Samoa during the mid-nineties, which led to a decline in taro production by over 70%, prompting some taro farmers to switch to other tuber crops [33].

This study was conducted to understand the prevalence of biotic stresses such as TLB and Dasheen mosaic virus (DsMV, genus *Potyvirus*), both of which significantly impact taro production [34,35]. The main objectives were as follows: (i) assess the socio-demographic characteristics of taro farmers; (ii) investigate the production practices by taro farmers; and (iii) estimate the incidence and severity of occurrence of TLB and DsMV in taro corms and leaves.

## 2. Results

### 2.1. Socio-Demographic Characteristics of Taro Farmers

The 63 taro farmers interviewed in the study included 41 males (65%) and 22 females (35%), aged 18 to 89 years, with an average of 49 years. From the responses of farmers, it was evident that most farmers (56%) have been cultivating taro for more than ten years, although it ranged from 2 to 45 years, indicating a high level of experience and familiarity with taro farming practices (Table 1). Table 1 illustrates the interrelationships among the farmers in the study. Oyo, Ekiti, Ondo, and Kwara States share cultural similarities and geographic boundaries. In these states, men predominantly engage in taro farming, comprising 21 of the 26 farmers surveyed in that location. In contrast, Akwa Ibom, Ebonyi, and Anambra States, which also share cultural similarities and boundaries, exhibit a higher representation of women in taro farming, with 16 of the 36 female farmers. Notably, these women have accumulated up to 20 years of experience in taro production.

Among the 53 taro fields assessed across seven states, 16 belonged to females (30%) and 37 to males (70%). Seventy-two percent of these fields were regular farms, while 28% were backyard farms, with mixed cropping as the predominant practice (79%). The size of regular farms ranged between 0.02 and 1.8 ha, while the portion of the farm allocated to taro ranged from 1 to 100%. The backyard farm sizes were ≤0.1 ha, and the portion of the farm allocated to taro was from 1 to 100%, with about 4 to 100 taro plants on the farm. Most of the farmers (63%) reported biotic stresses as the typical production constraint, followed by insufficient land (24%) for taro production (Table 2), with 44 farmers (70%) (Table 3) indicating TLB as a major constraint to taro production since 2009.

The survey results revealed that out of 44 farmers with TLB-infected plants, 41 of them (93%) did not use any management practices to control TLB, while two farmers from Ekiti used fungicide (Mancozeb) and one farmer from Oyo used sanitation to manage the disease. However, both farmers from Ekiti expressed dissatisfaction with the fungicide control due to the high cost. A total of 34 out of 44 farmers indicated a desire for resistant cultivars to control TLB. Two farmers, one from Oyo and the other from Kwara, reported that planting taro at the onset of the rainy season, rather than waiting for the rains to stabilize, helped reduce the severity of TLB. Additionally, all 63 farmers indicated that they reuse their planting materials from one season to another instead of using other available planting materials. The trend in production among farmers seems to show that 48% maintained the same level of output, while 35% experienced a decrease. Only 17% of farmers reported an increasing trend. The cumulative taro production from these farmers was 31,918 kg in 2017 and reduced to 31,264 kg in 2020 (Supplementary Table S1).

### 2.2. TLB Incidence

#### 2.2.1. Total Incidence

Out of 53 farms visited during the first survey, 44 (10 in Akwa Ibom, 9 in Ebonyi, 8 in Oyo, 6 in Anambra, 4 in Ekiti, 4 in Kwara, and 3 in Ondo) were found to have TLB infection, resulting in an overall prevalence of 83%. The incidence varied between 5% and 100%, with a median of 74.2%. The severity ranged from 1 to 2.5, with a median severity of 1.3. However, in the second survey, the prevalence was 25%, and the incidence ranged from 15% to 60% with a median of 20%. The severity also ranged from 1 to 1.8, with a median value of 1.0 (see Supplementary Table S2).

#### 2.2.2. TLB Incidence by Associated Ecosystems

##### Agroecosystems Associated with Derived Savanna (ADS)

A total of 18 out of 53 farms surveyed were situated in the ADS region. In the first survey, the TLB prevalence was found to be 89%; the incidence ranged between 5 and 100%, with a median of 80%, while the severity ranged from 1 to 2.5, with a median of 1.35. In the second survey, 10 out of 18 farms surveyed revealed a TLB prevalence of 50%, while the median incidence and severity were 20% and 1.0, respectively (Supplementary Table S3).

##### Agroecosystems Associated with Humid Forest (AHF)

A total of 35 out of 53 farms were in this region. During the first survey, the TLB prevalence was 80%, the incidence ranged between 10 and 100%, with a median of 65%, and the severity ranged from 1 to 1.9, with a median value of 1.3. During the second survey, TLB was not observed in 10 farms assessed, which showed zero prevalence and an incidence of 0% (Supplementary Table S4).

#### 2.2.3. TLB Incidence by Gene Pool

##### Farms with Dasheen Type

A total of 10 out of 53 farms had Dasheen taro, which showed 100% TLB prevalence; the incidence ranged between 5% and 100%, with a median of 82.3%. The severity of TLB ranged from 1 to 2.5 during the first survey, with a median of 1.4. In the second survey, nine farms were revisited, and the percentage prevalence was 56%, while the median incidence and severity were 20% and 1, respectively (Supplementary Table S5).

##### Farms with Eddoe Type

A total of 43 out of 53 farms had *Eddoe taro* plants and showed 79% TLB prevalence with 5 to 100% incidence, with a median of 67.5%. The severity ranged from 1 to 1.9 during the first survey, with a median of 1.3. Similarly, 11 farms revisited during the second survey showed zero TLB prevalence and incidence (Supplementary Table S6).

### 2.3. Incidence of Virus Diseases

**Figure 2:**
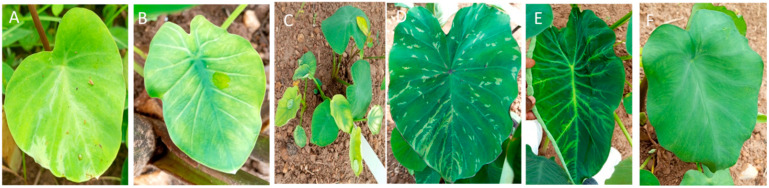
Virus symptoms shown by the collected samples. (**A**) shows deformation of leaves and chlorotic and mosaic patterns; (**B**) shows vein chlorosis; (**C**) shows severe stunting; (**D**,**E**) show mosaic patterns on the leaves; (**F**) shows leaf curling.

Viral disease-symptomatic plants were not observed in the fields surveyed. However, 70 plants regenerated from taro corms obtained from 19 farms and 8 markets. Of these, 33 were collected from seven farms and 37 were from five market lots. The observed symptoms were mosaic patterns, leaf chlorosis, deformation, and extreme stunting across the affected plants (Figure 2). The symptomatic plants showed 20% and 4.6% incidence of DsMV and generic *Potyvirus*, respectively (Table 4).

RT-PCR diagnostics detected DsMV in 69 of 70 symptomatic plants. None of the asymptomatic plants tested positive for the presence of DsMV or generic *Potyvirus* (Table 4). The harvested corms from the plants collected from 19 farms showed virus incidence of 27% and 7.4% based on the amplification with RT-PCR primers specific to DsMV and generic *Potyvirus*, respectively (Table 5), while the corms harvested from the plants collected from markets showed virus incidence of 16.1% and 3.1% for DsMV and generic *Potyvirus*, respectively (Table 6). All the plants that tested positive for generic *Potyvirus* were found to be positive for DsMV.

**Figure 3:**
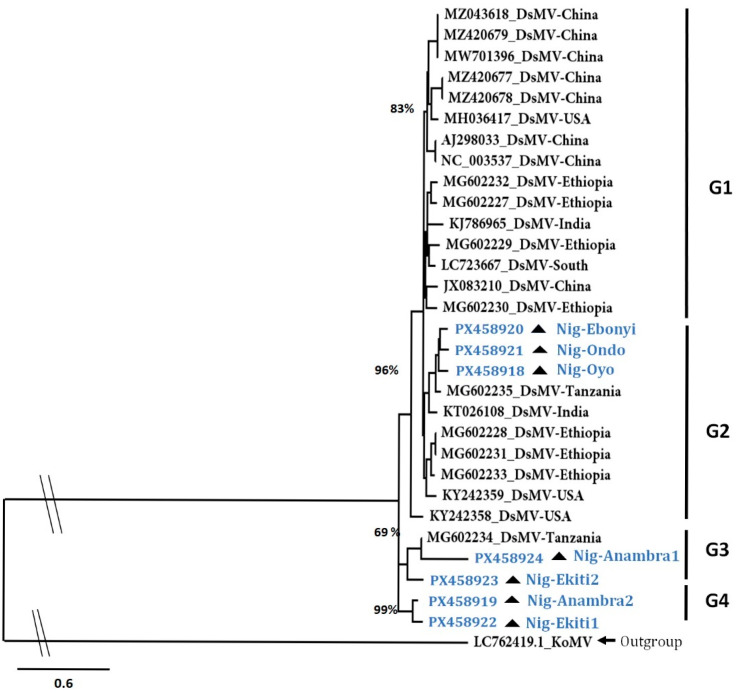
Diversity of the DsMV isolates from this study using the partial CI gene segment. The phylogenetic tree was constructed using the TN + F + I + G4 substitution model at 1000 bootstrap replicates. The Bootstrap branch support is indicated. The tree was constructed using the konjac mosaic virus isolate (LC762419.1) trimmed to the CI region as the outgroup. DsMV isolates from this study are indicated in blue font with the “Nig” prefix and the triangle symbol.

The sequencing of the DsMV partial cylindrical inclusion (CI) region obtained from the symptomatic taro plants revealed high sequence identity with DsMV isolates in the NCBI database (Supplementary Table S7). Phylogenetic reconstruction of DsMV diversity using the sequences of the isolates from the database, as well as from the samples of this study, showed four major groups: GI, G2, G3, and G4. The isolates in G1 and G2 originated from several countries spanning different continents, including Africa (Figure 3). The seven DsMV isolates sequenced in this study clustered in groups 2, 3, and 4, demonstrating a diversity within the DsMV isolates of Nigeria.

## 3. Discussion

Taro cultivation in Africa dates back to the 10th century [36], with its cultivation in Nigeria estimated to have started between 1965 and 1980 [14,15]. Historically, taro was perceived as a woman’s crop [1,37]. However, this study revealed that 65% of surveyed farmers were men. The study highlights the longevity of taro farming, with some farmers practicing for over four decades, suggesting that taro production relies on experienced farmers who possess in-depth knowledge of local agricultural conditions and effective traditional farming practices. The average age of the farmers interviewed was 49, indicating an aging yet experienced demographic. According to Jaji et al. [38] and Szabo et al. [39], older farmers are experienced but often reluctant to adopt new technologies and innovations in the control and management of plant diseases. Therefore, it is needful to encourage younger generations to engage in farming for sustainable taro production.

Taro production in Nigeria began to decline in 2008 [16]. The main purpose of this study was to consult with farmers to assess their perceived constraints to taro production and identify the reasons for the decline in production. Biotic stress was the primary production constraint, accounting for 63%, with TLB being the most significant biotic factor at 70%. Some localized studies have also revealed TLB as a major production constraint for taro [13,40,41]. The interviewed farmers noticed the onset of TLB in 2009, aligning with Bandyopadhyay et al.’s [19] first report of *P. colocasiae*’s association with the TLB epidemic in Nigeria that year. Out of the 63 farmers interviewed, 44 had TLB in their fields during the first survey, with only 5 implementing control measures, including adjusting planting times and using fungicides. Most farmers were unaware of TLB or its management practices. There is an urgent need to improve awareness about TLB to prevent a reduction in the quantity and quality of taro production in Nigeria.

The TLB has been linked to yield losses of 50–75% and the production of unpalatable corms [42]. According to the farmers interviewed, the stability of taro production varied over the years, with 48% indicating it was stable, 35% reporting a decline, and 17% noting an increase. This pattern coincides with the onset of TLB in Nigeria; however, the data do not demonstrate the quantitative link between national yield decline and TLB prevalence. Notably, 30% of farmers reported having TLB-free fields during the study. Cumulative production data from 2017 to 2020 showed an overall decline, highlighting the significant negative impact of TLB on taro production.

Most of the surveyed farms (79%) practiced mixed cropping, and a greater proportion of farms (70%) engaged in regular field farming compared to those that utilized backyard farming. This indicates a shift in taro production in Nigeria from a backyard activity to a more mainstream agricultural practice. However, the average sizes of the fields dedicated to taro production were quite small, with regular fields averaging 0.18 hectares and backyards averaging 0.02 hectares. This suggests that advanced farming practices, such as mechanization, are rarely feasible in Nigeria. Farmers could form groups to pool resources and enhance taro production, while jointly acquiring larger plots that are suitable for mechanization.

Precipitation and humidity are the key factors influencing the occurrence of TLB [43,44,45,46]. The first survey was conducted during the wet season (August–September), while the second survey was carried out during the dry season (December). The prevalence (83%), median incidence (74.2%), and severity (1.3) during the wet season survey were higher than those during the dry season survey, with a prevalence (25%), median incidence (20%), and severity (1.0) (Supplementary Table S2). During the dry season, TLB was found in flooded wetland farms, where standing water was 3–5 cm above the soil surface. This probably created the humidity necessary for the TLB pathogen to thrive. Additionally, the prevalence, incidence, and severity of TLB in ADS (89%, 80% and 1.35) (Supplementary Table S3) and AHF (80%, 65%; 1.3) (Supplementary Table S4) agroecosystems during the wet season revealed that TLB can thrive well in both environments, though the values were higher in ADS. However, Dasheens showed the highest TLB incidence, with a 100% prevalence rate. It is also noteworthy that Dasheens were only found in the derived savanna ecosystem, which had higher values of prevalence, incidence, and severity than the humid forest environment. This may be attributed to the fact that Dasheens were commonly planted in flooded wetlands, which provide a favorable condition for the TLB pathogen [47]. Another plausible explanation could be genetic differences; our recent study on Dasheens and Eddoes accessions from these environments revealed a profound divergence in population structure and molecular variance [48].

An assessment of viruses in corms collected from farms and markets revealed the presence of DsMV. Tests for other viruses, such as begomoviruses and badnaviruses, returned negative results. Additionally, the phylogenetic analysis of the DsMV isolates from Nigeria, compared with a representative set from the NCBI database, revealed that the Nigerian DsMV isolates are more closely related to those from Africa. The continuous recycling of planting materials from one season to the next, without the introduction of new seeds or improved varieties, makes controlling TLB and viral diseases difficult [38]. It is crucial to raise awareness about the risks associated with recycling farmers’ materials, and the need to provide regular training for farmers on good agricultural practices and quality seed production, including the selection of suitable planting materials and disease management strategies. Moreover, there is a need to strengthen taro seed systems to support the ongoing efforts as part of the Value-Added Crops program in Africa.

## 4. Limitations and Future Directions

While this study provides important baseline information on the prevalence of TLB and DsMV, we acknowledge limitations in the sampling design that may influence the interpretation of disease distribution. Survey sites were selected from areas of intensive taro cultivation and proximity to major commodity markets to ensure inclusion of regions with the largest production, rather than through a strictly stratified random sampling procedure. As such, the sample size of 63 farms should not be interpreted as fully representative at the national level, and no power analysis was performed to formally assess sample adequacy. Corms were collected opportunistically across farms and markets, resulting in variation in sample numbers between sites that could bias incidence estimates. The two surveys were conducted in different seasons (rainy vs. dry), which may also have introduced confounding effects on TLB incidence. Nonetheless, the findings provide a foundation for understanding the occurrence and distribution of the two most economically important diseases in major taro production zones. Future surveys with stratified random sampling across agroecosystems and multi-season assessments are necessary to strengthen epidemiological inference and guide regional management strategies.

## 5. Materials and Methods

### 5.1. Study Area

**Figure 4:**
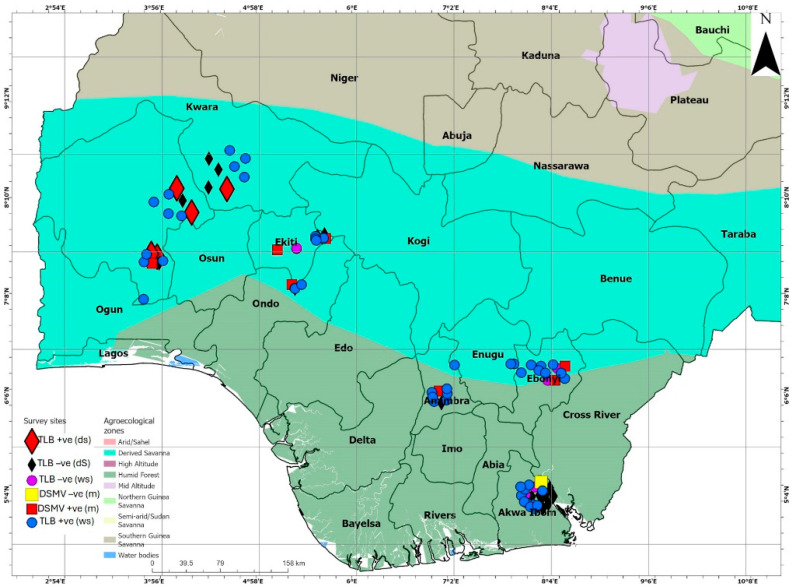
A map of Nigeria revealing agroecological zones, surveyed locations, and the status of taro leaf blight (TLB) in the surveyed areas in the wet (circles) and dry (triangles) seasons. It also shows the status of Dasheen mosaic virus (DsMV) in the market-sourced taro (square blocks). +ve = positive; -ve = negative. The markers on the map were spaced out for better visualization.

This study was conducted across 28 taro farming communities and ten markets in seven major taro-producing states (Oyo, Ekiti, Ondo, Kwara, Akwa-Ibom, Ebonyi, and Anambra) representing derived savanna and humid forest agroecosystems in Nigeria (Figure 4; Supplementary Table S8). A purposive sampling technique [49] was used to select 63 taro farmers and 26 marketers for this study. The data collection was performed in three separate surveys: between August and September 2021 (wet season covering 53 farms), between December 2021 and January 2022 (harvesting/dry season and covered 24 farms, a subset of the 53 farms assessed in the first survey, with the remaining farms having already been harvested) and between December 2021 and January 2022 (marketing season and covered eight markets).

### 5.2. Sampling Procedure and Data Collection

**Figure 5:**
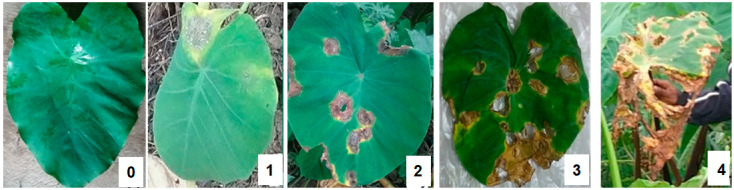
Images of leaves with varying TLB severity. 0 is a leaf with no TLB infection; 1 is a leaf with TLB infection within 1–25%; 2 is a leaf with TLB infection within 26–50%; 3 is a leaf with TLB infection within 51–75%; 4 is a leaf with TLB infection within 76–100%.

A structured questionnaire (Supplementary Document S9) was administered to collect the socio-demographic data of the farmers, including their gender, age, and knowledge of taro production, biotic constraints, and management practices. A separate survey form (Supplementary Document S10) was used to record the incidence and severity of foliar disease symptoms, especially those caused by *P. colocasiae* and virus. Educational materials, such as pictures of TLB and common taro viruses (mainly symptoms such as feathery and mosaic patterns, malformation and crinkling of the foliage, chlorosis, and severe stunting), were made available to farmers to facilitate the interview. Verbal consent was obtained from each participant before conducting the interviews. Additionally, data on the incidence and disease severity of TLB were also estimated from 20 plants in each field by using a “W” walking pattern and evaluating five plants along each transect of the “W” pattern [50]. The prevalence, severity, and incidence of TLB were calculated using the method of Abdulai et al. [46]. The severity rating was on a 0 to 4 rating scale, where 0 = no infection, 1 = 1–25% infection, 2 = 26–50% infection, 3 = 51–75% infection, and 4 = 76–100% infection (Figure 5). A total of 187 corms were collected from 19 out of 24 fields (harvesting time) for laboratory assessment of corm-borne diseases. In addition, 262 corms (all Eddoes) were collected from 26 marketers for laboratory-based studies on corm-borne diseases. The corms collected were local ecotypes.

### 5.3. Virus Assessment of Taro Corms

Corms collected from farmers’ fields (N = 187) and markets (N = 262) were planted in January 2022 at the IITA, Ibadan, Nigeria (N 7°29′56.562″ and E 3°54′27.5868″). Plants were assessed for symptoms at monthly intervals. The leaf samples of plants with typical Dasheen mosaic virus (DsMV, genus *Potyvirus*) symptoms were collected from fully grown plants and tested using DsMV-specific primers as well as generic primers to potyviruses using reverse transcription-polymerase chain reaction (RT-PCR). The same samples were also assessed by PCR with generic primers to detect begomoviruses, and appropriate virus-positive and negative (healthy) controls were used as assay controls. Primer sets used in this study have been previously validated for high sensitivity and specificity in detecting potyviruses and begomoviruses (Table 7).

### 5.4. Total Nucleic Acid (TNA) Extraction, Virus Detection by RT-PCR/PCR and Sequencing

The TNA from 100 mg taro leaf samples was extracted as per the protocol described by Ogunsola et al. [52]. Briefly, taro leaf tissues were crushed using a mortar and pestle in 1 mL of CTAB buffer, vortexed, and incubated at 60 °C for 10 min. An amount of 600 µL of phenol-chloroform-isoamyl alcohol (25:24:1) was added to the homogenized mixture. TNA was precipitated using isopropanol, and the pellet was washed twice with 500 µL of 70% (*v*/*v*) ethanol at 12,000 rcf for 5 min, and the pellet was air dried in an incubator set at 37 °C for 30 min. The pellet was suspended in 50 µL of nuclease-free water and stored at −20 °C until further use. The quantity and quality of isolated DNA were assessed using the NanoDrop ND2000 Spectrophotometer (Thermo Fisher Scientific Inc., Oxford, UK). To detect DsMV, 10 ng/µL of TNA was used following RT-PCR, while PCR/RT-PCR was used to detect potyviruses and begomoviruses using genus-specific primers (Table 7). RT-PCR/PCR reactions were performed in 12.5 µL reaction mixture comprising 10× PCR reaction buffer (supplied with Taq enzyme), 0.75 µL of 25 mM MgCl2, 0.25 µL mixture of 10 mM deoxynucleotide triphosphates (N0447L, New England Biolabs, Hertfordshire, UK), 0.25 µL of respective primers, 12 units of Moloney murine leukemia virus (M-MLV) reverse transcriptase (RT) (Promega Corporation, Madison, WI, USA), 0.3 units of Taq DNA polymerase (Promega Corporation, Madison, WI, USA), and 2.0 µL of 10 ng/µL total RNA and sterile distilled water. Virus RNA was amplified with a thermal cycler (Model XPRT, SeeAmp Thermal Cycler, Seegene, Seoul, Republic of Korea). Amplified RT-PCR/PCR products were separated in 1% agarose gel in 0.5× TBE buffer electrophoresis alongside a 100 bp DNA ladder and visualized under a UV transilluminator (BioRad GelDoc EZ Gel Imaging System, Bio-Rad Laboratories, Inc., Hercules, CA, USA) after staining in ethidium bromide (0.5 µg/mL). Samples with PCR amplicons showing expected amplification band sizes were purified through ethanol precipitation and further sequenced using the Sanger sequencing method at Iowa State University DNA Sequencing Facility (Ames, IA, USA). The DNA sequences of seven DsMV isolates generated in this study were deposited in the NCBI GenBank (Acc. No. PX458918 to PX458924).

### 5.5. Data Analyses

#### 5.5.1. Socio-Demographic Data

Data was collected based on interviews with selected farmers and visiting taro farms and markets, and observing taro production practices in the farms. Demographic details were captured about farmers (gender, age, and years of experience in taro production) and the farm (area under taro, occurrence of diseases and their symptoms (such as taro leaf blight and virus symptoms including feathery mosaic, leaf distortion, and stunting). Data was analyzed using a Microsoft Excel workbook (Office 365).

#### 5.5.2. TLB Disease Incidence and Severity

Incidence of TLB was estimated by counting the total number of TLB-infected plants and dividing it by the total number of plants observed. The severity of TLB for the plant was estimated by taking the total disease severity scores from the TLB-infected plants and dividing by the total number of TLB-infected plants.

% TLB Disease incidence = (Number of plants with TLB disease)/(Total number of Observed plants) × 100

% TLB Disease severity (Plant) = (Total of disease severity scores taken on TLB-infected plant)/(Total number of TLB-infected plants) × 100

#### 5.5.3. Virus Disease Incidence

The incidence of the disease was estimated from the number of plants with disease divided by the total number of emerged taro plants. This was calculated for each taro plant.

% Virus Disease incidence = (Number of plants with virus infection)/(Total number of emerged plants) × 100

#### 5.5.4. DsMV Phylogenetic Analysis

Sanger’s sequences from the virus-indexed samples were cleaned and assembled using BioEdit 7.2.5 [54] and compared with the NCBI database [non-redundant (nr)] using Blastn [55]. Based on the Blastn result, the evolutionary analysis was carried out on the cylindrical inclusion (CI) sequences by aligning them with complete genomic sequences of DsMV available in the NCBI database (accessed on 12 May 2023). The analysis also included konjac mosaic virus (KoMV) sequence as outgroup and was performed by using MAFFT software v7.0 (https://mafft.cbrc.jp/alignment/server/) (accessed on 12 May 2023) [56]. Aligned sequences were trimmed to about 400 nucleotides. A phylogenetic tree was constructed using aligned sequences in MEGA version 11 [57] following the neighbor-joining method with 1000 bootstrap replications.

## 6. Conclusions

Most of the surveyed taro farmers were male and relatively old but experienced in taro farming. Many were smallholder farmers and practiced mixed farming, although there has been a shift from backyard farming to regular field farming. TLB has been identified as one of the major biotic factors impeding taro production in Nigeria. This study confirmed that precipitation greatly influences TLB. Therefore, assessing the trend in weather conditions with TLB epidemiology will help make forecasts that can help mitigate disease impact and aid in creating sustainable disease management strategies. The type of field, upland (well-drained field) or flooded wetland, where taro is planted, also impacts the occurrence of TLB disease. Furthermore, the study reveals a heavy reliance of farmers on self-sourced planting materials, a reason for the spread of TLB disease. The phylogeny association of DsMV isolates in Nigeria with other DsMV isolates from Africa and beyond reveals the co-evolution of this virus. This is the first diagnostic confirmation of DsMV in Nigeria.

This study identified biotic factors as the major challenge to increased taro production in Nigeria. Addressing these challenges will require a multifaceted approach that includes enhanced extension services, improved access to certified planting materials, promoting sustainable agricultural practices, and supporting research and development to breed improved TLB and virus-resistant, high-yielding taro varieties. In addition, there is a need to develop market infrastructure, provide financial services, and strengthen farmer cooperatives for improved economic viability of taro farming in Nigeria.

## Figures and Tables

**Table 1:** Demographic data of the interviewed farmers.

State	Number of Farmers	Males	Females	Age Range (Years)	Years of Experience in Taro Cultivation (Years)	Median Year in Taro Cultivation
Oyo	9	7	2	40–70	5–40	25
Ekiti	8	7	1	32–65	5–31	10
Ondo	6	3	3	25–89	4–30	10
Kwara	4	4	0	55–70	8–15	10
Akwa Ibom	14	10	4	30–70	2–40	7
Ebonyi	15	10	5	18–65	8–45	5
Anambra	7	0	7	39–75	2–21	20
Total	63	41	22			

**Table 2:** Production constraints faced by the 63 farmers.

Production Constraints Encountered	No of Farmers	Percentage (%)
Pests and diseases	40	63
Insufficient land	15	24
Herdsmen crises	2	3.2
Erratic rainfall	3	5
Lack of money	4	6
Erosion and flooding	2	3.2
Herbicide unavailability	2	3.2
Scarce planting material	2	3.2
Lack of storage facility	2	3.2
Soil infertility	1	1.6
Labor cost	1	1.6
None	13	21

**Table 3:** Biotic constraints of taro as reported by the 63 farmers.

Pests and Diseases Encountered	No of Farmers	Percentage (%)
TLB	44	70
DsMV	2	3.2
Ants	1	1.6
None	18	29

**Table 4:** Incidence of virus diseases among the taro corms.

State	Number of Taro Corms Collected from Fields and Markets	Number Emerged	Number Symptomatic for Viruses	Positive with PCR (DsMV)	Positive with PCR (*Potyvirus*)
Oyo	35	20	3	3	1
Ekiti	138	113	27	27	5
Ondo	31	17	6	6	4
Kwara	20	3	1	1	0
Akwa Ibom	31	29	0	0	0
Ebonyi	128	105	16	16	0
Anambra	66	58	17	16	6
Total	449	345	70	69	16
Incidence (%)				20	4.6

**Table 5:** Incidence of virus disease among the corms collected from the fields.

State	LGA	Comm.	Taro GP	TCC/PCC	EP/VSP	DsMV + (PCR)	*Potyvirus* + (PCR)
Oyo	Akinyele	Idi Ose	Dasheen	5/5	1/0	0	0
Oyo	Akinyele	Sagbe	Dasheen	3/3	1/0	0	0
Oyo	Akinyele	Sagbe	Dasheen	6/6	3/2	2	0
Oyo	Ogbomoso North	Sabo	Dasheen	5/5	0/0	0	0
Oyo	Ogbomoso North	Sabo	Dasheen	1/1	0/0	0	0
Oyo	Ogbomoso North	Sabo	Eddoes	5/1	5/0	0	0
Ekiti	Ekiti East	Kota-Omuo	Eddoes	27/6	22/9	9	0
Ekiti	Ekiti East	Eda-Ile	Eddoes	30/6	27/10	10	5
Ekiti	Ekiti East	Eda-Ile	Eddoes	40/6	33/5	5	0
Ondo	Akure North	Bolorunduro	Eddoes	16/6	6/4	4	4
Ondo	Akure North	Bolorunduro	Eddoes	8/5	4/2	2	0
Kwara	Ilorin South	Oke Yalu	Dasheens	5/5	1/1	1	0
Kwara	Ilorin South	Oke Yalu	Dasheens	5/5	1/1	0	0
Kwara	Ilorin South	Oke Yalu	Dasheens	5/5	0	0	0
Kwara	Ilorin South	Oke Yalu	Dasheens	5/5	1/1	0	0
Akwa Ibom	Itu	Ikot Ekpuk	Eddoes	1/1	1/1	0	0
Akwa Ibom	Itu	Ikot Ekpuk	Eddoes	5/1	5/0	0	0
Akwa Ibom	Ibiono Ibom	Ikot idaha	Eddoes	5/1	5/0	0	0
Anambra	Idemili North	Uke	Eddoes	10/2	6/0	0	0
	Total			187/77	122/33	33	9
	Incidence (%)				27(VSP)	27	7.4

LGA: Local government area; Comm.: Community; GP: Gene pool; TCC: Total corms collected; PCC: Plants from where corms were collected; EP: Emerged plants; VSP: Virus-symptomatic plants; DsMV + (PCR): Plants positive for DsMV in PCR; *Potyvirus* + (PCR): Plants positive for *Potyvirus* in PCR.

**Table 6:** Incidence of viral disease among the corms collected from the markets.

State	LGA	Market	STS	TCC/EP	VSP	DsMV + (PCR)	*Potyvirus* + (PCR)
Oyo	Ibadan North	Bodija	Anambra and Enugu	10/10	1	1	1
Ekiti	Ado	Oja Oba	Ekiti	10/6	2	2	0
Ekiti	Ado	Oja Oba	Ekiti	8/5	1	1	0
Ekiti	Ado	Oja Oba	Ekiti	6/5	0	0	0
Ekiti	Ado	Oja Oba	Ekiti	10/10	0	0	0
Ekiti	Ekiti East	Kota-Omuo	Ekiti	7/5	0	0	0
Ondo	Akure North	Ogbese	Ondo	7/7	0	0	0
Akwa Ibom	Itu	Itu	Cross River	10/10	0	0	0
Akwa Ibom	Itu	Itu	Cross River	10/8	0	0	0
Ebonyi	Izzi	Iboko	Ebonyi	10/9	6	6	0
Ebonyi	Izzi	Iboko	Ebonyi	8/7	0	0	0
Ebonyi	Izzi	Iboko	Ebonyi	10/8	0	0	0
Ebonyi	Izzi	Iboko	Ebonyi	10/6	1	1	0
Ebonyi	Izzi	Iboko	Ebonyi	10/10	0	0	0
Ebonyi	Izzi	Iboko	Ebonyi	10/10	2	2	0
Ebonyi	Abakaliki	International	Ebonyi	10/10	1	1	0
Ebonyi	Abakaliki	International	Ebonyi and Cross River	20/17	3	3	0
Ebonyi	Abakaliki	International	Ebonyi	10/9	1	1	0
Ebonyi	Abakaliki	International	Ebonyi and Cross River	20/13	1	1	0
Ebonyi	Abakaliki	International	Cross River	10/6	1	1	0
Anambra	Idemili South	Eke	Anambra	10/10	6	6	2
Anambra	Idemili South	Eke	Anambra	10/10	2	2	1
Anambra	Idemili South	Eke	Anambra	6/6	1	1	0
Anambra	Idemili South	Eke	Anambra	10/10	4	4	1
Anambra	Idemili South	Eke	Anambra	10/10	1	1	0
Anambra	Idemili South	Eke	Anambra	10/6	3	2	2
	Total			262/223	37	36	7
	Incidence (%)				16.6	16.1	3.1

LGA: Local government area; STS: Source of corm sold; TCC: Total corms collected; EP: Emerged plants; VSP: Virus symptomatic plants; DsMV + (PCR): Plants positive for DsMV on PCR; *Potyvirus* + (PCR): Plants positive for *Potyvirus* on PCR.

**Table 7:** Details of primers and PCR/RT-PCR conditions used for the detection of viruses in taro leaf samples.

Virus and Amplicon Size (bp)	Primer (5′ to 3′)	Cycle Conditions	Reference
DsMV (447)	DsMV-F: CAGGCACATCAATTTTTCTAA	1 × 30 min at 42 °C, 1 × 94 °C for 3 min, and 35× at 94 °C for 30 s, 52 °C for 30 s, 72 °C for 1 min; 1 × 72 °C for 5 min	[51]
	DsMV-R: GGCTCCACACCATAAATGTGCACG		
*Potyvirus* [Cylindrical Inclusion (CI)] (683)	CI-F: CGIVIGTIGGIWSIGGIAARTCIAC3	1 × 42 °C for 30 min, 1 × 94 °C for 2 min and 35 × at 94 °C for 1 min, 50 °C for 1 min, 72 °C for 1 min; 1 × 72 °C for 5 min	[52]
	CI-R: ACICCRTTYTCDATDATRTTIGTIGC3		
Begomovirus (520)	PA: TAATATTACCKGWKGVCCSC	1 × 94 °C for 3 min, and 35× at 94 °C for 30 s, 50 °C for 30 s, 72 °C for 1 min; 1 × 72 °C for 5 min	[53]
	PB: TGGACYTTRCAWGGBCCTTCACA		

## Data Availability

The data underlying this article are available in the article.

## Supplementary Materials

The following supporting information can be downloaded at: https://www.mdpi.com/article/10.3390/plants14223457/s1, Table S1: Taro production of the 63 farmers from 2017 to 2020 in kg; Table S2: Incidence and severity of TLB across the farms during the first and second survey; Table S3: TLB incidence and severity in the derived savanna agroecosystem; Table S4: TLB incidence and severity in the humid forest agroecosystem; Table S5: TLB incidence among farms with Dasheen taro; Table S6: TLB incidence in farms with Eddoe taro; Table S7: Reference table of NCBI sequences outside this study used to run the DsMV virus phylogenetic tree; Table S8: Description of the study area; Document S9: Questionnaire; Document S10: Taro Leaf Blight (TLB) Survey Protocol, cited in [58]; Figure S1: Disease symptoms on taro leaves; Figure S2: Field layouts and monitoring survey and sample collection path; Figure S3: Field layout and sample collection, cited in [58].

## Abbreviations

These are the abbreviations used in the manuscript:

ADS	Agroecosystem associated with derived savanna
AHF	Agroecosystem associated with humid forest
TLB	Taro leaf blight
TNA	Total nucleic acid
DsMV	Dasheen mosaic virus
PCR	Polymerase chain reaction
CI	Cylindrical inclusion
M-MLV	Moloney murine leukemia virus
RT	Reverse transcriptase
RT-PCR	Reverse transcription polymerase chain reaction
DNA	Deoxyribonucleic acid
RNA	Ribonucleic acid
UV	Ultraviolet
KoMV	Konjac mosaic virus
nr	Non-redundant
NCBI	National Center for Biotechnology Information
MAFFT	Multiple Alignment using Fast Fourier Transform
MEGA	Molecular Evolutionary Genetics Analysis

## References

[1] Onyeka J. (2014). Status of Cocoyam (Colocasia esculenta and Xanthosoma spp.) in West and Central Africa: Production, Household Importance and the Threat from Leaf Blight.

[2] Oladimeji J.J., Kumar P.L., Abe A., Vetukuri R.R., Bhattacharjee R. (2022). Taro in west Africa: Status, challenges, and opportunities. Agronomy.

[3] Rashmi D.R., Raghu N., Gopenath T.S., Palanisamy P., Bakthavatchalam P., Karthikeyan M., Gnanasekaran A., Ranjith M.S., Chandrashekrappa G.K., Basalingappa K.M. (2018). Taro (*Colocasia esculenta*): An overview. J. Med. Plants Stud..

[4] Chaïr H., Traore R.E., Duval M.F., Rivallan R., Mukherjee A., Aboagye L.M., Van Rensburg W.J., Andrianavalona V., Pinheiro de Carvalho M.A.A., Saborio F. (2016). Genetic Diversification and Dispersal of Taro (*Colocasia esculenta* (L.) *Schott*). PLoS ONE.

[5] Kreike C.M., Van Eck H.J., Lebot V. (2004). Genetic diversity of taro, *Colocasia esculenta* (L.) Schott, in Southeast Asia and the Pacific. Theor. Appl. Genet..

[6] Grimaldi I.M., van Andel T.R. (2018). Food and medicine by what name? The ethnobotanical and linguistic diversity of Taro in Africa. Econ. Bot..

[7] Nair U.M.A., Devi A.A., Veena S.S., Krishnan P., Arya R.S. (2018). Genetic Diversity Analysis of Leaf Blight Resistant and Susceptible Taro [*Colocasia esculenta* (L.) Schott] Genotypes Using ISSR Markers. J. Root Crops.

[8] Maretta D., Helianti I., Santosa E. (2022). Sustainability cultivation and traditional conservation of taro diversity in Bogor Indonesia. IOP Conf. Ser. Earth Environ. Sci..

[9] Hossain M.M., Asaduzzaman M., Khan M.A., Akter L., Ismail T., Akhtar S., Lazarte C.E. (2023). Taro (*Colocasia* spp.): Applications in Food Production and Improving Nutrition in South Asia. Neglected Plant Foods of South Asia: Exploring and Valorizing Nature to Feed Hunger.

[10] Temesgen M., Retta N. (2015). Nutritional potential, health and food security benefits of taro *Colocasia esculenta* (L.): A review. Food Sci. Qual. Manag..

[11] Aditika, Kapoor B., Singh S., Kumar P. (2021). Taro (*Colocasia esculenta*): Zero wastage orphan food crop for food and nutritional security. S. Afr. J. Bot..

[12] Mitharwal S., Kumar A., Chauhan K., Taneja N.K. (2022). Nutritional, phytochemical composition and potential health benefits of taro (*Colocasia esculenta* L.) leaves: A review. Food Chem..

[13] Fufa T.W., Oselebe H.O., Nnamani C.V., Afiukwa C.A., Uyoh E.A. (2021). Systematic Review on Farmers’ Perceptions, Preferences, and Utilization Patterns of Taro [*Colocasia esculenta* (L.) Schott] for Food and Nutrition Security in Nigeria. J. Plant Sci..

[14] Ubalua A.O., Ewa F., Okeagu O.D. (2016). Potentials and challenges of sustainable taro (*Colocasia esculenta*) production in Nigeria. J. Appl. Biol. Biotechnol..

[15] Akinrinola T., Tijani-Eniola H. (2025). The growth and yield of cocoyam (*Colocasia esculenta* (L.) *Schott*) Affected By Storage methods. Rev. Fac. Nac. Agron. Medellín.

[16] FAOSTAT FAO Statistical Database 2025. https://www.fao.org/faostat/en/#data/QCL.

[17] Fufa T.W., Oselebe H.O., Abtew W.G., Amadi C. (2023). Physicochemical analysis of taro (*Colocasia esculenta* (L.) Schott) accessions. Asian J. Res. Agric. For..

[18] Kumar R. (2019). Genetic variability among taro (*Colocasia esculenta* L. Schott Var. antiquorum) Germplasm related to morphological and nutritional characters: A review. J. Pharmacogn. Phytochem..

[19] Bandyopadhyay R., Sharma K., Onyeka T.J., Aregbesola A., Kumar P.L. (2011). First Report of Taro (*Colocasia esculenta*) Leaf Blight Caused by *Phytophthora colocasiae* in Nigeria. Plant Dis..

[20] Vetukuri R.R., Kushwaha S.K., Sen D., Whisson S.C., Lamour K.H., Grenville-Briggs L.J. (2018). Genome Sequence Resource for the Oomycete Taro Pathogen *Phytophthora colocasiae*. Mol. Plant Microbe Interact..

[21] Raciborski M. (1900). Parasitic algae and fungi, Java. Batavia Bull. N. Y. State Mus..

[22] Ooka J.J. (1990). Taro diseases. Research-Extension-Series. Hawaii Inst. Trop. Agric. Hum. Res..

[23] Brasier C., Scanu B., Cooke D., Jung T. (2022). *Phytophthora*: An ancient, historic, biologically and structurally cohesive and evolutionarily successful generic concept in need of preservation. IMA Fungus.

[24] Hieno A., Li M., Feng W., Afandi A., Otsubo K., Suga H., Kageyama K. (2022). Detection and identification of *Phytophthora* pathogens that are threatening forest ecosystems worldwide. River Basin Environment: Evaluation, Management and Conservation.

[25] Carnot A.C., Emmanuel E.L., Ledoux N.D.G., Patrick A.N., Arthur M.J., Annie N.N., Zachée A., Fabrice M.T., Gautier D.L. (2017). Study of antagonistic beneficial microorganisms to *Phytophtora colocasiae*, causal agent of taro mildew (*Colocasia esculenta* (L.) Schott). Plant.

[26] Padmaja G., Devi G.U., Mahalakshmi B.K., Sridevi D. (2017). Characterization of Isolates of *Phytophthora colocasiae* Collected from Andhra Pradesh and Telangana Causing Leaf Blight of Taro. Int. J. Curr. Microbiol. Appl. Sci..

[27] Jackson G.V.H., Gollifer D.E. (1975). Storage rots of taro (*Colocasia esculenta*) in the British Solomon Islands. Ann. Appl. Biol..

[28] Mbong G.A., Fokunang C.N., Manju E.B., Njukeng A.P., Tembe-Fokunang E.A., Hanna R. (2015). Mycelia growth and sporulation of *Phytophthora colocasiae* isolates under selected conditions. Am. J. Exp. Agric..

[29] Adomako J., Kwoseh C., Moses E. (2017). Metalaxyl sensitivity and aggressiveness of *Phytophthora colocasiae* isolates associated with taro leaf blight disease. Plant Pathol. J..

[30] Tchameni S.N., Mbiakeu S.N., Sameza M.L., Jazet P.M.D., Tchoumbougnang F. (2018). Using *Citrus aurantifolia* essential oil for the potential biocontrol of *Colocasia esculenta* (taro) leaf blight caused by *Phytophthora colocasiae*. Environ. Sci. Pollut. Res..

[31] Moïse N.A.A., Severin T.N., Christelle S.E., Tibo A.A.H., Lambert S.M., Duplex W.J. (2018). Efficacy of *Trichoderma harzianum* (Edtm) and *Trichoderma aureoviride* (T4) as a potential bio-control agent of taro leaf blight caused by *Phytophthora colocasiae*. Int. J. Appl. Microbiol. Biotechnol..

[32] Islam W. (2018). Plant Disease Epidemiology: Disease Triangle and Forecasting Mechanisms in Highlights. Hosts Viruses.

[33] Hunter D., Pouono K., Semisi S. (1998). The impact of taro leaf blight in the Pacific Islands with special reference to Samoa. J. South Pac. Agric..

[34] Nelson S.C. (2008). Dasheen mosaic of edible and ornamental aroids. Plant Dis..

[35] Kidanemariam D.B., Sukal A.C., Abraham A.D., Njuguna J.N., Stomeo F., Dale J.L., James A.P., Harding R.M. (2021). Incidence of RNA viruses infecting taro and tannia in East Africa and molecular characterisation of dasheen mosaic virus isolates. Ann. Appl. Biol..

[36] Grimaldi I.M., Leke W.N., Borokini I., Wanjama D., Van Andel T. (2018). From landraces to modern cultivars: Field observations on taro *Colocasia esculenta* (L.) Schott in sub-Saharan Africa. Genet. Resour. Crop Evol..

[37] Opata P.I., Ogbonna P.E. (2015). Storage profitability and effectiveness of storage methods in yield loss reduction in cocoyam in southeast Nigeria. Afr. J. Agric. Res..

[38] Jaji F.O., Yusuf-Oshoala M.A., Issa F.O. (2013). Root and Tuber Expansion Program Technologies and Farmers’ Productivity in Lagos and Ogun States of Nigeria. OIDA Int. J. Sustain. Dev..

[39] Szabo S., Apipoonyanon C., Pramanik M., Tsusaka T.W., Leeson K. (2021). Agricultural productivity, aging farming workforce, sustainable agriculture, and well-being: Household survey data from Central Thailand. Front. Sustain. Food Syst..

[40] Ayogu C.J., Ike C.U., Ogbonna O.I., Nnaemeka G.K. (2015). Agricultural Extension Roles towards adapting to the effects of taro leaf blight (TLB) disease in Nsukka Agricultural Zone, Enugu State. J. Biol. Agric. Healthc..

[41] Shutt V.M., Mwanja P.Y., Affiah D.U., Edward M.O. (2022). Occurrence of leaf blight of cocoyam (*Colocasia esculenta*) caused by *Phytophthora colocasiae* in Jos East LGA, Plateau State, Nigeria. Afr. Phytosanit. J..

[42] Nelson S., Brooks F., Teves G. (2011). Taro Leaf Blight in Hawai‘i. Plant Dis..

[43] Lakshmi B.K.M., Reddy R.V.S.K., Babu D. (2012). Impact of Weather Parameters on the Incidence of Leaf Blight Disease in Taro (*Colocasia esculenta* (L.) Schott.). J. Root Crops.

[44] Singh D., Jackson G., Hunter D., Fullerton R., Lebot V., Taylor M., Okpul T., Tyson J. (2012). Taro leaf blight—A threat to food security. Agriculture.

[45] Shakywar R.C., Pathak S.P., Mahesh P., Tomar K.S., Hem S. (2013). Developmental behaviour of leaf blight of taro caused by *Phytophthora colocasiae*. Vegetos.

[46] Abdulai M., Norshie P.M., Santo K.G. (2020). Incidence and severity of taro (*Colocasia esculenta* L.) blight disease caused by *Phytophthora colocasiae* in the Bono Region of Ghana. Int. J. Agric. Environ. Sci..

[47] Otieno C.A. (2020). Taro leaf blight (*Phytophthora colocasiae*) disease pathogenicity on selected taro (*Colocasiae esculenta*) accessions in Maseno, Kenya. OALib.

[48] Oladimeji J.J., Abe A., Kumar P.L., Agre P.A., Ilesanmi O.J., Vetukuri R.R., Bhattacharjee R. (2024). Extent and patterns of morphological and molecular genetic diversity and population structure of Nigerian Taro cultivars. BMC Plant Biol..

[49] Boivin G., Sauriol P. (1984). Dispersion statistics and sequential sampling plan for leaf blight caused by *Botrytis squamosa* in onions [Quebec]. Phytopathology.

[50] Palinkas L.A., Horwitz S.M., Green C.A., Wisdom J.P., Duan N., Hoagwood K. (2015). Purposeful sampling for qualitative data collection and analysis in mixed method implementation research. Adm. Policy Ment. Health Ment. Health Serv. Res..

[51] Wang Y., Wu B., Borth W.B., Hamim I., Green J.C., Melzer M.J., Hu J.S. (2017). Molecular characterization and distribution of two strains of Dasheen mosaic virus on Taro in Hawaii. Plant Dis..

[52] Ogunsola K.E., Ilori C., Fatokun C.A., Boukar O., Ogunsanya P., Kumar P.L. (2021). Disease incidence and severity in cowpea lines evaluated for resistance to single and multiple infections of endemic viruses in Nigeria. J. Crop Improv..

[53] Deng D., McGrath P.F., Robinson D.J., Harrison B.D. (1994). Detection and differentiation of whitefly-transmitted geminiviruses in plant and vector insects by the polymerase chain reaction with degenerate primers. Ann. Appl. Biol..

[54] Hall T.A. (1991). BioEdit: A user-friendly biological sequence alignment editor and analysis program for Windows 95/98/NT. Nucleic Acids Symp. Ser..

[55] Zhang Z., Schwartz S., Wagner L., Miller W. (2000). A greedy algorithm for aligning DNA sequences. J. Comput. Biol..

[56] Katoh K., Rozewicki J., Yamada K.D. (2019). MAFFT online service: Multiple sequence alignment, interactive sequence choice and visualization. Brief Bioinform..

[57] Kumar S., Stecher G., Li M., Knyaz C., Tamura K. (2018). MEGA X: Molecular Evolutionary Genetics Analysis across computing platforms. Mol. Biol. Evol..

[58] Virology and Molecular Diagnostic Unit, International Institute of Tropical Agriculture (VMD, IITA) (2014). Baseline Survey on Banana Bunchy Top Disease (BBTD) Situation—Pilot Sites. BBTD Containment and Recovery: Building Capacity and Piloting Field Recovery Approaches Through a Learning Alliance.

