# Supplementary material for: Understanding Biotic Constraints to Taro (Colocasia esculenta) Production in the Derived Savanna and Humid Forest Agroecosystems of Nigeria
Source: Plants (Basel). 2025 Nov 12;14(22):3457. doi: 10.3390/plants14223457 (PMC12656383; doi:10.3390/plants14223457)
Supplement: Supplementary file 1 [file plants-14-03457-s001.zip › plants-3872456-supplementary.pdf]

Supplementary Table S1

Taro production of the 63 farmers from 2017 to 2020 in kg

SN	State	Farmer ID	Gender	2017	2018	2019	2020
1	Oyo	Akinyele 1	Male	20	20	20	20
2	Oyo	Akinyele 2	Male	50	50	50	50
3	Oyo	Akinyele 3	Male	10	10	10	10
4	Oyo	Oluyole 1	Male	10	10	10	10
5	Oyo	Ogbomoso North 1	Male	30	30	30	27
6	Oyo	Ogbomoso North	Male	10	9	8.5	9
7	Oyo	Ogbomoso North 3	Male	250	250	200	200
8	Oyo	Ogbomoso North 4	Female	300	300	300	300
9	Oyo	Ogbomoso South 1	Female	100	100	100	90
10	Ekiti	Ekiti East 1	Male	350	300	300	300
11	Ekiti	Ekiti East 2	Male	550	500	470	490
12	Ekiti	Ekiti East 3	Female	500	550	600	550
13	Ekiti	Ekiti East 4	Male	600	650	700	700
14	Ekiti	Ekiti East 5	Male	400	350	350	350
15	Ekiti	Gbonyin 1	Male	200	100	50	50
16	Ekiti	Gbonyin 2	Male	450	500	550	550
17	Ekiti	Gbonyin 3	Male	2500	2500	3000	3000
18	Ondo	Akure North 1	Male	5	5	4	2

19	Ondo	Akure North 2	Male	500	470	480	470
20	Ondo	Akure North	Male	3500	4000	3000	2500
21	Ondo	Akure North	Female	2500	2600	3000	3000
22	Ondo	Akure North	Female	4500	3000	3500	4000
23	Ondo	Owo 1	Female	2500	2000	2000	1800
24	Kwara	Ilorin South 1	Male	200	150	100	120
25	Kwara	Ilorin South 2	Male	5000	5000	5000	5000
26	Kwara	Ilorin South 3	Male	200	180	150	150
27	Kwara	Ilorin South 4	Male	30	25	30	30
28	Akwa Ibom	Itu 1	Female	180	150	100	100
29	Akwa Ibom	Itu 2	Female	0	5	5	5
30	Akwa Ibom	Itu 3	Male	10	9	8	7
31	Akwa Ibom	Itu 4	Male	200	150	120	110
32	Akwa Ibom	Itu 5	Female	0	0	300	250
33	Akwa Ibom	Uyo 1	Male	100	80	50	50
34	Akwa Ibom	Uyo 2	Female	150	150	120	100
35	Akwa Ibom	Uyo 3	Male	5	4	3	3

36	Akwa Ibom	Uyo 4	Male	5	4	3	4
37	Akwa Ibom	Uyo 5	Male	4	6	5	5
38	Akwa Ibom	Ibiono Ibom 1	Male	5	2	2	1
39	Akwa Ibom	Ibiono Ibom 2	Male	1000	900	870	950
40	Akwa Ibom	Ibiono Ibom 3	Male	2	1	1	1
41	Akwa Ibom	Ibiono Ibom 4	Male	2	1	0.5	0.5
42	Ebonyi	Ohaukwu 1	Male	350	400	350	370
43	Ebonyi	Ohaukwu 2	Female	200	150	170	180
44	Ebonyi	Ohaukwu 3	Female	0	0	75	80
45	Ebonyi	Ohaukwu 4	Female	350	500	480	550
46	Ebonyi	Ohaukwu 5	Male	200	250	270	290
47	Ebonyi	Ohaukwu 6	Male	600	700	700	800
48	Ebonyi	Ezza North 1	Male	1000	1200	1100	1300
49	Ebonyi	Ezza North 2	Female	200	100	50	80
50	Ebonyi	Ezza North 3	Male	0	0	110	110
51	Ebonyi	Ezza North 4	Male	0	450	500	450
52	Ebonyi	Abakaliki 1	Male	100	110	120	110
53	Ebonyi	Abakaliki 2	Male	50	50	60	55

54	Ebonyi	Abakaliki 3	Female	100	100	100	100
55	Ebonyi	Abakaliki 4	Male	100	120	80	70
56	Ebonyi	Abakaliki 5	Male	25	30	25	25
57	Anambra	Idemili North 1	Female	1200	1000	1100	900
58	Anambra	Idemili North 2	Female	35	50	45	40
59	Anambra	Idemili North 3	Female	50	48	50	50
60	Anambra	Idemili North 4	Female	100	90	85	75
61	Anambra	Idemili North 5	Female	120	100	70	65
62	Anambra	Idemili North 6	Female	60	65	50	50
63	Anambra	Idemili North 7	Female	150	145	150	150
			Total	31918	30779	31340	31264

Supplementary Table S2

Incidence and severity of TLB across the farms during the first and second survey

			Survey 1			Survey 2		
State	LGA	Community	Farm number	TLB Incidence (%)	Mean severity of TLB plants	Farm number	TLB Incidence (%)	Mean severity of TLB plants
Oyo	Akinyele	Idi Ose	1	80	1.1	1	50	1.8
Oyo	Akinyele	Sagbe	2	75	1.3	2	0	0
Oyo	Akinyele	Sagbe	3	5	1	3	15	1
Oyo	Oluyole	Idi Ayunre	4	5	1	4	-	-
Oyo	Ogbomoso North	Sabo	5	20	1.5	5	10	1
Oyo	Ogbomoso North	Sabo	6	95	1.3	6	60	1.4
Oyo	Ogbomoso North	Sabo	7	85	1.3	7	0	0
Oyo	Ogbomoso South	Sun-unsun-un	8	5	1	8	-	-
Ekiti	Ekiti East	Kota-Omuo	9	80	1.4	9	Dried	Dried
Ekiti	Ekiti East	Eda-Ile	10	80	1.4	10	-	-
Ekiti	Ekiti East	Eda-Ile	11	100	1.4	11	Dried	Dried
Ekiti	Ekiti East	Eda-Ile	12	75	1.2	12	Dried	Dried
Ekiti	Gbonyin	Ilu Omooba	13	0	0	13	-	-
Ekiti	Gbonyin	Ilu Omooba	14	0	0	14	-	-
Ondo	Akure North	Bolorunduro	15	40	1.5	15	0	0

Ondo	Akure North	Bolorunduro	16	100	1.75	16	0	0
Ondo	Owo	Amure	17	100	1.75	17	-	-
Kwara	Ilorin South	Oke Yalu	18	100	1.75	18	0	0
Kwara	Ilorin South	Oke Yalu	19	85	1.8	19	0	0
Kwara	Ilorin South	Oke Yalu	20	100	1.6	20	10	1
Kwara	Ilorin South	Oke Yalu	21	20	2.5	21	0	0
Akwa Ibom	Itu	Nwtusiong	22	100	1.4	22	0	0
Akwa Ibom	Itu	Ikot Ekpuk	23	10	1	23	0	0
Akwa Ibom	Itu	Ikot Ekpuk	24	0	0	24	0	0
Akwa Ibom	Itu	Ikot Ekpuk	25	75	1.4	25	0	0
Akwa Ibom	Itu	Itam	26	100	1.9	26	-	-
Akwa Ibom	Uyo	Nung obio enang	27	100	1.7	27	0	0
Akwa Ibom	Uyo	Nung obio enang	28	60	1	28	0	0
Akwa Ibom	Uyo	Nung obio enang	29	80	1.3	29	-	-
Akwa Ibom	Uyo	Nung obio enang	30	0	0	30	-	-

Akwa Ibom	Ibiono Ibom	Ikot Idaha	31	35	1	31	0	0
Akwa Ibom	Ibiono Ibom	Ikot Idaha	32	95	1.9	32	0	0
Akwa Ibom	Ibiono Ibom	Ikot Idaha	33	0	0	33	-	-
Akwa Ibom	Ibiono Ibom	Ikot Idaha	34	75	1	34	-	-
Ebonyi	Ohaukwu	Ntuskpa	35	100	1.8	35	-	-
Ebonyi	Ohaukwu	Ntuskpa	36	75	1.3	36	-	-
Ebonyi	Ohaukwu	Nwankuu Ntsurakpa Izhia	37	25	1.2	37	-	-
Ebonyi	Ohaukwu	Umueghara	38	65	1.4	38	-	-
Ebonyi	Ohaukwu	Ezzangwu Amaechi	39	0	0	39	-	-
Ebonyi	Ezza North	Oshiegbe	40	0	0	40	-	-
Ebonyi	Ezza North	Oshiegbe	41	90	1.9	41	-	-
Ebonyi	Ezza North	Umu ezeohohoa	42	35	1.7	42	-	-
Ebonyi	Abakaliki	Agbaja Azumili	43	65	1	43	-	-
Ebonyi	Abakaliki	Agbaja Azumili	44	50	1.6	44	-	-
Ebonyi	Abakaliki	Unagboke	45	0	0	45	-	-
Ebonyi	Abakaliki	Unagboke	46	10	1	46	-	-

Ebonyi	Abakaliki	Egugwu Agbaja unhufu	47	0	0	47	-	-
Anambra	Idemili North	Uke	48	50	1.3	48	Dried	Dried
Anambra	Idemili North	Uke	49	45	1.3	49	-	-
Anambra	Idemili North	Uke	50	60	1.1	50	-	-
Anambra	Idemili North	Uke	51	35	1.1	51	-	-
Anambra	Idemili North	Uke	52	70	1.2	52	-	-
Anambra	Idemili North	Uke	53	35	1	53	-	-
Median TLB incidence and severity				74.2%	1.3		25%	1.0

LGA: Local government area

-: Farms not visited during the second survey because they had completed their harvesting

Supplementary Table S3

TLB incidence and severity in the derived savanna agroecosystem

			Survey 1			Survey 2		
State	LGA	Community	Farm numbers	TLB Incidence (%)	Mean severity of TLB plants	Farm numbers	TLB Incidence (%)	Mean severity of TLB plants
Oyo	Akinyele	Idi Ose	1	80	1.1	1	50	1.8
Oyo	Akinyele	Sagbe	2	75	1.3	2	0	0
Oyo	Akinyele	Sagbe	3	5	1	3	15	1
Oyo	Oluyole	Idi Ayunre	4	5	1	4	-	-
Oyo	Ogbomoso North	Sabo	5	20	1.5	5	10	1
Oyo	Ogbomoso North	Sabo	6	95	1.3	6	60	1.4
Oyo	Ogbomoso North	Sabo	7	85	1.3	7	0	0
Oyo	Ogbomoso South	Sun-unsun-un	8	5	1	8	-	-
Ekiti	Ekiti East	Kota-Omuo	9	80	1.4	9	Dried	Dried
Ekiti	Ekiti East	Eda-Ile	10	80	1.4	10	-	-
Ekiti	Ekiti East	Eda-Ile	11	100	1.4	11	Dried	Dried
Ekiti	Ekiti East	Eda-Ile	12	75	1.2	12	Dried	Dried
Ekiti	Gbonyin	Ilu Omooba	13	0	0	13	-	-
Ekiti	Gbonyin	Ilu Omooba	14	0	0	14	-	-

Kwara	Ilorin South	Oke Yalu	15	100	1.75	15	0	0
Kwara	Ilorin South	Oke Yalu	16	85	1.8	16	0	0
Kwara	Ilorin South	Oke Yalu	17	100	1.6	17	10	1
Kwara	Ilorin South	Oke Yalu	18	20	2.5	18	0	0
Median TLB incidence and severity				80%	1.35		20%	1.0

LGA: Local government area

-: Farms not visited during the second survey because they had completed their harvesting

Supplementary Table S4

TLB incidence and severity in the humid forest agroecosystem

			Survey 1			Survey 2		
State	LGA	Community	Farm number	TLB Incidence (%)	Mean severity of TLB plants	Farm number	TLB Incidence (%)	Mean severity of TLB plants
Ondo	Akure North	Bolorunduro	1	40	1.5	1	0	0
Ondo	Akure North	Bolorunduro	2	100	1.75	2	0	0
Ondo	Owo	Amure	3	100	1.75	3	-	-
Akwa Ibom	Itu	Nwtusiong	4	100	1.4	4	0	0
Akwa Ibom	Itu	Ikot Ekpuk	5	10	1	5	0	0
Akwa Ibom	Itu	Ikot Ekpuk	6	0	0	6	0	0
Akwa Ibom	Itu	Ikot Ekpuk	7	75	1.4	7	0	0
Akwa Ibom	Itu	Itam	8	100	1.9	8	-	-
Akwa Ibom	Uyo	Nung obio enang	9	100	1.7	9	0	0
Akwa Ibom	Uyo	Nung obio enang	10	60	1	10	0	0
Akwa Ibom	Uyo	Nung obio enang	11	80	1.3	11	-	-

Akwa Ibom	Uyo	Nung obio enang	12	0	0	12	-	-
Akwa Ibom	Ibiono Ibom	Ikot Idaha	13	35	1	13	0	0
Akwa Ibom	Ibiono Ibom	Ikot Idaha	14	95	1.9	14	0	0
Akwa Ibom	Ibiono Ibom	Ikot Idaha	15	0	0	15	-	-
Akwa Ibom	Ibiono Ibom	Ikot Idaha	16	75	1	16	-	-
Ebonyi	Ohaukwu	Ntuskpa	17	100	1.8	17	-	-
Ebonyi	Ohaukwu	Ntuskpa	18	75	1.3	18	-	-
Ebonyi	Ohaukwu	Nwankuu Ntsurakpa Izhia	19	25	1.2	19	-	-
Ebonyi	Ohaukwu	Umueghara	20	65	1.4	20	-	-
Ebonyi	Ohaukwu	Ezzangwu Amaechi	21	0	0	21	-	-
Ebonyi	Ezza North	Oshiegbe	22	0	0	22	-	-
Ebonyi	Ezza North	Oshiegbe	23	90	1.9	23	-	-
Ebonyi	Ezza North	Umu ezeohohoa	24	35	1.7	24	-	-
Ebonyi	Abakaliki	Agbaja Azumili	25	65	1	25	-	-
Ebonyi	Abakaliki	Agbaja Azumili	26	50	1.6	26	-	-

Ebonyi	Abakaliki	Unagboke	27	0	0	27	-	-
Ebonyi	Abakaliki	Unagboke	28	10	1	28	-	-
Ebonyi	Abakaliki	Egugwu Agbaja unhufu	29	0	0	29	-	-
Anambra	Idemili North	Uke	30	50	1.3	30	Dried	Dried
Anambra	Idemili North	Uke	31	45	1.3	31	-	-
Anambra	Idemili North	Uke	32	60	1.1	32	-	-
Anambra	Idemili North	Uke	33	35	1.1	33	-	-
Anambra	Idemili North	Uke	34	70	1.2	34	-	-
Anambra	Idemili North	Uke	35	35	1	35	-	-
Median TLB incidence and severity				65%	1.3		0%	0

LGA: Local government area

-: Farms not visited during the second survey because they had completed their harvesting

Supplementary Table S5

TLB incidence among farms with Dasheen taro

			Survey 1			Survey 2		
State	LGA	Community	Farm number	TLB Incidence (%)	Mean severity of TLB plants	Farm number	TLB Incidence (%)	Mean severity of TLB plants
Oyo	Akinyele	Idi Ose	1	80	1.1	1	50	1.8
Oyo	Akinyele	Sagbe	2	75	1.3	2	0	0
Oyo	Akinyele	Sagbe	3	5	1	3	15	1
Oyo	Ogbomoso North	Sabo	4	20	1.5	4	10	1
Oyo	Ogbomoso North	Sabo	5	95	1.3	5	60	1.4
Oyo	Ogbomoso South	Sun-unsun-un	6	5	1	6	-	-
Kwara	Ilorin South	Oke Yalu	7	100	1.75	7	0	0
Kwara	Ilorin South	Oke Yalu	8	85	1.8	8	0	0
Kwara	Ilorin South	Oke Yalu	9	100	1.6	9	10	1
Kwara	Ilorin South	Oke Yalu	10	20	2.5	10	0	0
Median TLB incidence and severity				82.3%	1.4		20%	1

LGA: Local government area

-: Farms not visited during the second survey because they had completed their harvesting

Supplementary Table S6

TLB incidence in farms with Eddoe taro

			Survey 1			Survey 2		
State	LGA	Community	Farm number	TLB Incidence (%)	Mean severity of TLB plants	Farm number	TLB Incidence (%)	Mean severity of TLB plants
Oyo	Oluyole	Idi Ayunre	1	5	1	1	-	-
Oyo	Ogbomoso North	Sabo	2	85	1.3	2	0	0
Ekiti	Ekiti East	Kota-Omuo	3	80	1.4	3	Dried	Dried
Ekiti	Ekiti East	Eda-Ile	4	80	1.4	4	-	-
Ekiti	Ekiti East	Eda-Ile	5	100	1.4	5	Dried	Dried
Ekiti	Ekiti East	Eda-Ile	6	75	1.2	6	Dried	Dried
Ekiti	Gbonyin	Ilu Omooba	7	0	0	7	-	-
Ekiti	Gbonyin	Ilu Omooba	8	0	0	8	-	-
Ondo	Akure North	Bolorunduro	9	40	1.5	9	0	0
Ondo	Akure North	Bolorunduro	10	100	1.75	10	0	0
Ondo	Owo	Amure	11	100	1.75	11	-	-
Akwa Ibom	Itu	Nwtusiong	12	100	1.4	12	0	0
Akwa Ibom	Itu	Ikot Ekpuk	13	10	1	13	0	0
Akwa Ibom	Itu	Ikot Ekpuk	14	0	0	14	0	0

Akwa Ibom	Itu	Ikot Ekpuk	15	75	1.4	15	0	0
Akwa Ibom	Itu	Itam	16	100	1.9	16	-	-
Akwa Ibom	Uyo	Nung obio enang	17	100	1.7	17	0	0
Akwa Ibom	Uyo	Nung obio enang	18	60	1	18	0	0
Akwa Ibom	Uyo	Nung obio enang	19	80	1.3	19	-	-
Akwa Ibom	Uyo	Nung obio enang	20	0	0	20	-	-
Akwa Ibom	Ibiono Ibom	Ikot Idaha	21	35	1	21	0	0
Akwa Ibom	Ibiono Ibom	Ikot Idaha	22	95	1.9	22	0	0
Akwa Ibom	Ibiono Ibom	Ikot Idaha	23	0	0	23	-	-
Akwa Ibom	Ibiono Ibom	Ikot Idaha	24	75	1	24	-	-
Ebonyi	Ohaukwu	Ntuskpa	25	100	1.8	25	-	-
Ebonyi	Ohaukwu	Ntuskpa	26	75	1.3	26	-	-
Ebonyi	Ohaukwu	Nwankuu Ntsurakpa Izhia	27	25	1.2	27	-	-
Ebonyi	Ohaukwu	Umueghara	28	65	1.4	28	-	-
Ebonyi	Ohaukwu	Ezzangwu Amaechi	29	0	0	29	-	-
Ebonyi	Ezza North	Oshiegbe	30	0	0	30	-	-

Ebonyi	Ezza North	Oshiegbe	31	90	1.9	31	-	-
Ebonyi	Ezza North	Umu ezeohohoa	32	35	1.7	32	-	-
Ebonyi	Abakaliki	Agbaja Azumili	33	65	1	33	-	-
Ebonyi	Abakaliki	Agbaja Azumili	34	50	1.6	34	-	-
Ebonyi	Abakaliki	Unagboke	35	0	0	35	-	-
Ebonyi	Abakaliki	Unagboke	36	10	1	36	-	-
Ebonyi	Abakaliki	Egugwu Agbaja unhufu	37	0	0	37	-	-
Anambra	Idemili North	Uke	38	50	1.3	38	Dried	Dried
Anambra	Idemili North	Uke	39	45	1.3	39	-	-
Anambra	Idemili North	Uke	40	60	1.1	40	-	-
Anambra	Idemili North	Uke	41	35	1.1	41	-	-
Anambra	Idemili North	Uke	42	70	1.2	42	-	-
Anambra	Idemili North	Uke	43	35	1	43	-	-
Median TLB incidence and severity				67.5%	1.3		0%	0

LGA: Local government area

-: Farms not visited during the second survey because they had completed their harvesting

Supplementary Table S7

Reference table of NCBI sequences outside this study used to run the DsMV virus phylogenetic tree

Accession	Virus	Crop	Country	Sequence length	Sequence amplified	Reference
JX083210	DsMV	Blue Dragon	China	10030	Complete genome	Liu and Li, 2012
MG602232	DsMV	Cocoyam	Ethiopia	10027	Complete genome	Kidanemariam, 2018
MG602230	DsMV	Taro	Ethiopia	10036	Complete genome	Kidanemariam, 2018
KJ786965	DsMV	Elephant foot yam	India	10024	Complete genome	Kamala et al., 2014
MG602233	DsMV	Cocoyam	Ethiopia	10073	Complete genome	Kidanemariam, 2018
MG602227	DsMV	Taro	Ethiopia	10073	Complete genome	Kidanemariam, 2018
MG602228	DsMV	Taro	Ethiopia	10032	Complete genome	Kidanemariam, 2018
MG602231	DsMV	Cocoyam	Ethiopia	10056	Complete genome	Kidanemariam, 2018
KY242358	DsMV	Taro	USA	10002	Complete genome	Wang et al., 2016
KY242359	DsMV	Taro	USA	10019	Complete genome	Wang et al., 2016
AJ298033	DsMV	Arum lily	China	10038	Complete genome	Chen et al., 2001
MG602229	DsMV	Taro	Ethiopia	10037	Complete genome	Kidanemariam, 2018

NC_003537	DsMV	Arum lily	China	10038	Complete genome	Chen et al., 2001
MG602234	DsMV	Cocoyam	Tanzania	10075	Complete genome	Kidanemariam, 2018
KT026108	DsMV	Taro	India	10004	Complete genome	Liebrecht and Winter, 2015
MG602235	DsMV	Cocoyam	Uganda	10033	Complete genome	Kidanemariam, 2018
MW701396	DsMV	Giant voodoo lily	China	9737	ss-RNA	Qin et al., 2021
MZ043618	DsMV	Giant voodoo lily	China	9737	Complete genome	Qin et al., 2021
LC723667	DsMV	Calla lilies	South Korea	10007	Complete genome	Cho et al., 2022
MH036417	DsMV		USA	407	CI protein gene	Wang et al., 2018
MZ420678	DsMV		China	9834	Polyprotein gene, partial cds	Qin et al., 2021
MZ420677	DsMV		China	9826	Polyprotein gene, partial cds	Qin et al., 2021
MZ420679	DsMV		China			Qin et al., 2021

LC762419.1(outgroup)	KoMV	East Asian wildparsley	China	9286	complete genome	Park et al., 2023

Supplementary Table S8

Description of the study area

Agroecology	State	LGA	Community/ Market	Latitude	Longitude	Altitude (m)	No of farmers/ Marketers interviewed	# of farms visited	# of farms revisited
Derived savanna	Oyo	Akinyele	Idi Ose	7.508	3.913	262.11	1	1	1
Derived savanna	Oyo	Akinyele	Sagbe	7.493	3.917	222.85	2	2	2
Derived savanna	Oyo	Oluyole	Idi Ayunre	7.213	3.856	137.05	1	1	-
Derived savanna	Oyo	Ogbomoso North	Sabo	8.147	4.239	350.97	4	3	3
Derived savanna	Oyo	Ogbomoso South	Sun-unsun-un	8.105	4.235	346.33	1	1	-
Derived savanna	Ekiti	Ekiti East	Ilasa	7.758	5.722	562.49	1	1	1
Derived savanna	Ekiti	Ekiti East	Eda Ile	7.749	5.643	565.18	4	3	2
Derived savanna	Ekiti	Gbonyin	Ilu omooba	7.632	5.421	419.96	3	2	-
Derived savanna	Kwara	Ilorin South	Oke Yalu	8.459	4.587	337.45	4	4	4
Derived savanna	Kwara	Asa	Ago	4.585	8.388	353.56	0	0	0

Humid Forest	Ondo	Akure North	Bolorunduro	7.221	5.402	335.24	2	2	2
Humid Forest	Ondo	Akure North	Ogbese	7.26	5.374	349.39	3	0	0
Humid Forest	Ondo	Owo	Amure	7.26	5.476	323.38	1	1	-
Humid Forest	Akwa Ibom	Itu	Nwtusiong	5.054	7.904	78.06	1	1	1
Humid Forest	Akwa Ibom	Itu	Ikot Ekpuk	5.057	7.904	92.2	3	3	2
Humid Forest	Akwa Ibom	Itu	Itam	5.055	7.916	93.96	1	1	-
Humid Forest	Akwa Ibom	Uyo	Nung obio enang	5.043	7.879	0	5	4	2
Humid Forest	Akwa Ibom	Ibiono Ibom	Ikot Idaha	5.041	7.873	82.91	4	4	3
Humid Forest	Ebonyi	Ohaukwu	Ntuskpa	6.432	7.693	124.36	3	2	-
Humid Forest	Ebonyi	Ohaukwu	Nwankuu Ntsurakpa Izhia	6.42	7.874	123.56	1	1	-
Humid Forest	Ebonyi	Ohaukwu	Umueghara	6.42	7.071	124.05	1	1	-
Humid Forest	Ebonyi	Ohaukwu	Ezzangwu Amaechi	6.41	7.974	130.58	1	1	-

Humid Forest	Ebonyi	Ezza North	Oshiegbe	6.364	7.952	116.67	2	2	-
Humid Forest	Ebonyi	Ezza North	Umu ezeohohoa	6.338	8.023	114.71	2	1	-
Humid Forest	Ebonyi	Abakaliki	Agbaja Azumili	6.324	8.13	70.1	2	2	-
Humid Forest	Ebonyi	Abakaliki	Unagboke	6.329	8.132	63.89	2	2	-
Humid Forest	Ebonyi	Abakaliki	Egpumpkin Agbaja unhufu	6.336	8.127	75.29	1	1	-
Humid Forest	Anambra	Idemili North	Uke	6.092	6.917	214.35	7	6	1
Markets									
Derived savanna	Oyo	Ibadan North	Bodija	7.434	3.911	227	1		
Derived savanna	Ekiti	Ado	Oja Oba	7.622	5.222	438	4		
Derived savanna	Ekiti	Ado	Okesa	7.617	5.223	190	0		
Derived savanna	Ekiti	Ekiti East	Kota-Omuo	7.758	5.722	539	1		
Humid Forest	Ondo	Akure North	Ogbese	7.26	5.374	349	1		
Humid Forest	Akwa Ibom	Itu	Itu	5.2	7.977	8	2		

Humid Forest	Akwa Ibom	Uyo	Plaza	5.027	7.977	6	0
Humid Forest	Ebonyi	Izzi	Iboko	6.408	8.226	78	6
Humid Forest	Ebonyi	Abakaliki	International	6.31	8.124	40	5
Humid Forest	Anambra	Idemili South	Eke	6.109	6.921	161	6

Supplementary Document S9

Questionnaire

Questionnaire Identification Number: ……………..

PART A: INTERVIEW BACKGROUND (Adapted from Baseline Survey on Banana Bunchy Top Disease (BBTD) Situation [58]

1. Date of interview: Day: ……………. Month: …………. Year: 20……

2. Locational Details

Country	State/ Region	LGA/ District	Village/ Community	Rainfall	Humidity	GPS readings of homestead			
						Waypoint ID	Latitude	Longitude	Altitude

3. Respondent’s name: …………………………………….. 4. Gender: M or F (**Circle**); 5. Age: ……. (**In years**)

6. Any telephone for contact: ………………………………………………………………………………………

PART B: TARO PRODUCTION PATTERNS AND CONSTRAINTS

1. How long have you been growing taro? …………….. (**In years**)

2. Over the past five years, what is your taro production trend? …. (**1=Decreasing; 2=About the same; 3=Increasing**)

3. What are the five major production constraints? Rank them in order of importance

(**starting by 1=the most important**)

Major constraints	Rank		

4. What pests and diseases are present and when do you encounter them? (**Use codes below**)

Pests and diseases encountered	When? (code A)	Pests and diseases control strategies if any

Code A: 1. Before corm development; 2: After corm development

5. Will you be interested to destroy all the diseased taros and replant with new plants? 1=Yes; 2=No and Reason

…………………………………………………….

6. What is the main source of your planting materials? … (**1=Own corms/cormels; 2=Purchased corms/cormels; 3=Other, specify**: ……..…….)

7. If purchased, from where? ……… (**1=Inside this village; 2=Outside the village**)

8. Did you get any problem of pest and diseases in your taro production last season?…1=Yes; 2=No

9. If answer is 'yes' for question 8, did you use chemicals or other methods to control it?...........

10. Were you satisfied with the control method?..... 1=Yes; 2=No

11. If answer is 'no' for question 10, state reason.................................

12. Are you aware of Taro Leaf Blight (TLB)? 1=Yes; 2=No

13. Are your fields affected by TLB? …… YES=1 NO=2; if yes from when (approximate date) and how much area was lost?.....................

14. Are you aware of any TLB control technologies? How did you get this information?

………
……

15. If you are aware of any TLB control technology but have not adopted any, what is the most important reason for non-use? (Circle one only)

1. Gathering more information about the technology

2. Technology not available
3. Too risky to adopt
4. Traditional control practice is better
5. Lack of cash
6. Lack of sufficient labour
7. Others (e.g. cultural factors)
   ……………………………………………………………………………

20. In case of non-adoption of TLB control technology, do you expect to adopt it later? .... YES=1 NO=2

21. If NO from 20 above, do you plan, never to adopt it? .... YES=1 NO=2

22. If YES from 21 above, give the reason for thinking never to adopt it
…………………………………………………………………

23. Which of the following methods do you use to control pests and diseases in your taro farm when you notice them?

Methods	Yes	No	Frequency of Usage			
			Not at all	Rarely	Occasionally	Regularly
Cultural						
Biological						
Chemical						
Indigenous						
Others, specify:						

4. What is the roughly estimated quantity of the taro corms you get from your farms in the past 4 years?

Farm size (ha)	Year 2017	Year 2018	Year 2019	Year 2020

Supplementary Document S10

Taro Leaf Blight (TLB) Survey Protocol

**Sheet #**		**Type of survey**	**Detection**	**Delimiting**	**Monitoring**
**Date/Time**		**Field size**			
**Location name**		**Age of plantation**			
**District / LGA**		***Colocasia esculenta* varieties**			
**State/Province**					
**Agro-ecology**		**Intercrops/crops around the field**			
**Latitude**					
**Longitude**		**Managed or unmanaged**			
**Altitude (m)**		**Weediness**			
		**Researcher (farmer)**			

**Field Summary:**		**Percent infection:**		**Severity range:**			
**Plant #**	**TLB Symptoms**	**Variety**	**Photo ID (if any)**	**DsMV**	**CBDV**	**TaBV**	**Observations/Comments**
1							
2							
3							
4							
5							
6							
7							
8							
9							
10							
11							
12							
13							
14							
15							
16							
17							
18							
19							
20							
**General observations/comments:**							

(Adapted from BBTD Survey and Surveillance Protocol [58])

Abbreviations for symptom description and severity score:

1. TLB Symptoms' description
   ns = no symptoms**; pl** = presence of lesions**; cs** = coalesce of spot**; cp** = collapse of the petiole
2. Virus disease scoring
   1= present; 0 = absent [Dasheen mosaic virus (DsMV), Colocasia bobone disease virus (CBDV), Taro bacilliform virus (TaBV)]

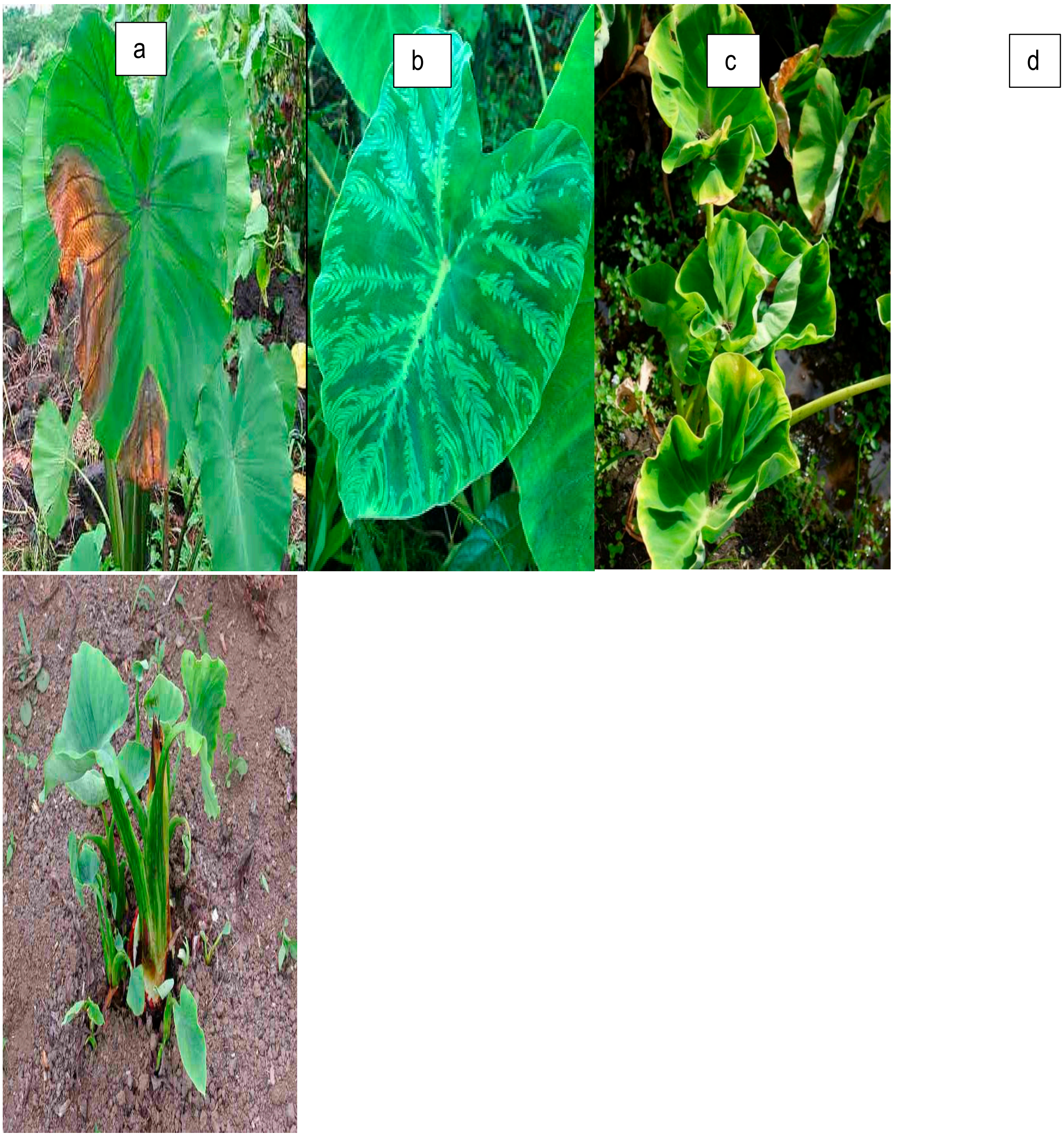

Figure S1: Disease symptoms on taro leaves (a = TLB; b = DsMV; c = CBDV; d = Alomae disease) (Bing images', n.d.)

<u>I. Sample collection and estimation of disease incidence in a field:</u> (Adapted from BBTD Survey and Surveillance Protocol, [58]

a) Monitoring survey (disease present and at high incidence)

In each field, take observations from 20 plants at random by walking across a ‘W’ shaped path, with 5 plants per side spaced at an equal distance from each other (Figure 2). Record symptom type and severity score. Collect 4 samples of taro blight symptomatic leaves for *Phytophthora colocasiae* isolation and testing (detailed below), sampling the first symptomatic plant (if one is present) from the 5 that are observed in each traverse. If no symptomatic plants are present, sample the last of the 5 observed.

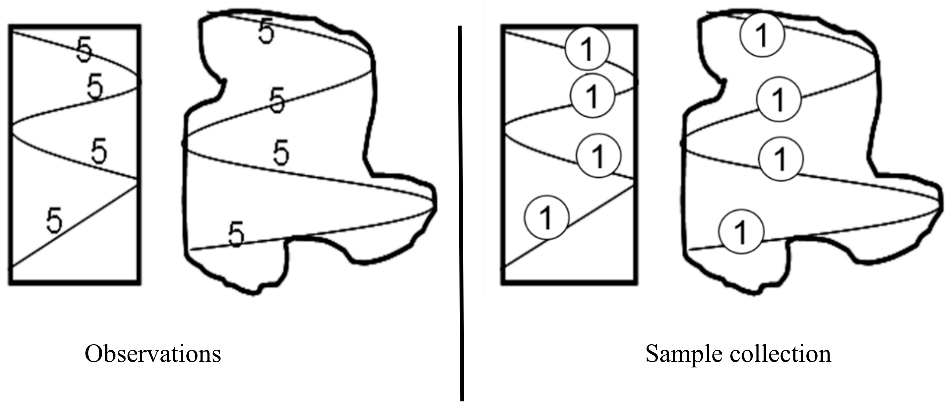

Figure S2. Field layouts and monitoring survey and sample collection path.

b) Sample collection for Microbiome study on soil and leaves

i. Leaf sample collection

In each field, take leaf samples from 20 at random by walking across a ‘W’ shaped path, with 5 plants (without disease symptoms) per side spaced at an equal distance from each other (Figure 3).

The collected leaves should be kept in paper bags or wrapped with paper cushions and kept in plastic bags

ii. Soil sample collection

In each field, dig to a depth of 15cm at a 5 cm distance from the main stem of the five plants at the "W" junction and collect soil samples using a soil auger. The collected soil from the rhizosphere of these plants is bulked to form a composite sample for the field.

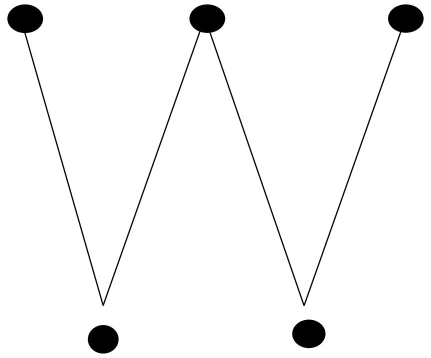

Sample collection

Figure S3. Field layout and sample collection **(Adapted from BBTD Survey and Surveillance Protocol, Kumar [57])**

II. TLB Symptom severity score and estimation of disease severity in a field:

Severity score, Disease severity (DS), and Disease incidence (DI) estimation				
S/N	Severity Score	DS and DI estimation	Type of Research	References

	5 Rating Score<br>**0** = no symptom; **1** = low infection (1-25% infection on leaf); **2** = moderate infection (26-50% infection on leaf); **3** = high infection (51-75% infection on leaf); **4** = very high infection (>75% infection on leaf)		Taro field survey to assess the incidence and severity of TLB	[45]

References

58. Virology and Molecular Diagnostic Unit, International Institute of Tropical Agriculture (VMD, IITA). Baseline Survey on Banana Bunchy Top Disease (BBTD) Situation – Pilot sites. BBTD containment and recovery: Building capacity and piloting field recovery approaches through a learning alliance. 2014.
